# Mycorrhizal-induced calmodulin mediated changes in antioxidant enzymes and growth response of drought-stressed trifoliate orange

**DOI:** 10.3389/fmicb.2014.00682

**Published:** 2014-12-05

**Authors:** Yong-Ming Huang, A. K. Srivastava, Ying-Ning Zou, Qiu-Dan Ni, Yu Han, Qiang-Sheng Wu

**Affiliations:** ^1^College of Horticulture and Gardening/Institute of Root Biology, Yangtze UniversityJingzhou, China; ^2^National Research Centre for CitrusNagpur, India

**Keywords:** CaM, Cu/Zn-SOD, drought stress, Mn-SOD, mycorrhizal fungi, ROS, trifoliate orange

## Abstract

Trifoliate orange [*Poncirus trifoliata* (L) Raf.] is considered highly arbuscular mycorrhizal (AM) dependent for growth responses through a series of signal transductions in form of various physiological responses. The proposed study was carried out to evaluate the effect of an AM fungus (*Funneliformis mosseae*) on growth, antioxidant enzyme (catalase, CAT; superoxide dismutase, SOD) activities, leaf relative water content (RWC), calmodulin (CaM), superoxide anion ($O_{2}^{•-}$), and hydrogen peroxide (H_2_O_2_) concentrations in leaves of the plants exposed to both well-watered (WW) and drought stress (DS) conditions. A 58-day of DS significantly decreased mycorrhizal colonization by 60% than WW. Compared to non-AM seedlings, AM seedlings displayed significantly higher shoot morphological properties (plant height, stem diameter, and leaf number), biomass production (shoot and root fresh weight) and leaf RWC, regardless of soil water status. AM inoculation significantly increased CaM and soluble protein concentrations and CAT activity, whereas significantly decreased $O_{2}^{•-}$ and H_2_O_2_ concentration under both WW and DS conditions. The AM seedlings also exhibited significantly higher Cu/Zn-SOD and Mn-SOD activities than the non-AM seedlings under DS but not under WW, which are triggered by higher CaM levels in AM plants on the basis of correlation studies. Further, the negative correlation of Cu/Zn-SOD and Mn-SOD activities with $O_{2}^{•-}$ and H_2_O_2_ concentration showed the DS-induced ROS scavenging ability of CaM mediated SODs under mycorrhization. Our results demonstrated that AM-inoculation elevated the synthesis of CaM in leaves and up-regulated activities of the antioxidant enzymes, thereby, repairing the possible oxidative damage to plants by lowering the ROS accumulation under DS condition.

## INTRODUCTION

Arbuscular mycorrhizal fungi (AMF) as an ubiquitously beneficial soil microorganism can build symbiotic association with citrus plant roots, popularly known as arbuscular mycorrhizas (AMs). Earlier study demonstrated a key role of AM in protecting host plants against detrimental effects of drought stress (DS; Wu et al., 2013). In recent past, massive efforts have been undertaken to study how the water deficit stress signals are perceived and transduced by the plants to activate the antioxidant pathways. Enhancement in drought tolerance of AM-inoculated plants is by and large reported to be governed by the nature of antioxidant protective system (Wu et al., 2006a, 2013; Wu and Zou, 2009), especially under DS conditions.

Drought stress is the most important abiotic factor, invariably limiting plant growth and yield in a variety of irrigated crops including citrus (Abbaspour et al., 2012; Wu et al., 2013). Generation and elimination of reactive oxygen species (ROS) in plants remain in dynamic balance under well-watered (WW) condition, but such balance of ROS is interrupted under DS condition, thereby, inducing an elevation in ROS concentration (Bowler et al., 1994). These ROS mainly comprise of superoxide anion $O_{2}^{•-}$, hydrogen peroxide (H_2_O_2_), and hydroxyl radical (HO⋅). An excessive accumulation of these ROS in cells can cause oxidative damage, through the processes involving lipids peroxidation, protein oxidation, DNA fragmentation, etc. (Sharma et al., 2010, 2012). As a consequence, plants also develop a complex enzymatic and non-enzymatic antioxidant protective system to scavenge overproduced ROS, thus alleviating the oxidative damage to plants (Sharma et al., 2012). Superoxide dismutases (SODs) are considered as the first line of defense against ROS catalyzing dismutation reaction of $O_{2}^{•-}$ into H_2_O_2_, and O_2_. H_2_O_2_ (an important signal transduction molecule and toxic byproduct) can then be scavenged by catalase (CAT). According to different metal atoms combined in SODs, they exist in three isoforms comprising copper/zinc SOD (Cu/Zn-SOD), manganese SOD (Mn-SOD), and iron SOD (Fe-SOD), which are located in different subcellular compartments (Bowler et al., 1994), with differential activities as per plant species.

Calmodulin (CaM) as an acidic protein is one of the best characterized Ca^2+^ receptors (Yang et al., 2010). CaM consists of two globular domains, each harboring a pair of EF-hands that can bind Ca^2+^, upon exposure of hydrophobic surfaces, develops high affinity binding sites for downstream effectors (Perochon et al., 2011). Although CaM has no enzymatic activity of its own, the binding of Ca^2+^ to CaM can activate numerous downstream target proteins. Ca^2+^/CaM complex as the messenger system, modulates a series of physiological and biochemical processes to reduce oxidative damage (Kim et al., 2009). Plants possess an interesting and rapidly growing list of CaM targets (Snedden and Fromm, 2001), including metabolic enzymes, transcription factors, etc. (Reddy and Reddy, 2004; Bouché et al., 2005). Roles of CaM in plant growth and development, besides fighting against stresses, such as salt damage, freezing injury and disease, are well documented (Yang and Poovaiah, 2003; Bouché et al., 2005; Du and Poovaiah, 2005; Hu et al., 2007). Nevertheless, there is hardly any information available highlighting the relationship of CaM with antioxidant enzymes under mycorrhization, especially under DS conditions. Likewise, the effect of AM on the relationship between CaM and SOD isoforms is poorly understood under DS. In this background, the present study was undertaken with two objectives: (i) analyze the effect of AMF, (*Funneliformis mosseae*), on relative water content (RWC), CaM concentration, SODs (Cu/Zn-SOD and Mn-SOD) activities, and ROS ($O_{2}^{•-}$ and H_2_O_2_) levels in leaves of trifoliate orange [*Poncirus trifoliata* (L.) Raf.] seedlings under WW and DS conditions and (ii) analyze the relationship between CaM and antioxidant enzymatic protective system under mycorrhization.

## MATERIALS AND METHODS

### PLANT CULTURE

Seeds of trifoliate orange (*Poncirus trifoliata* L. Raf.) were first surface-sterilized with 70% alcohol for 5 min, rinsed five times with distilled water, and germinated in autoclaved (0.11 Mpa, 121°C, 2 h) sands in a growth chamber (26/20°C day/night temperature, 740 μmol/m^2^/s photosynthetic photon flux density and 80% relative humidity). Twenty-three days later, seedlings (three four-leaf-old) were transferred to a plastic pot (15 cm upper diameter × 12 cm height × 10 cm bottom diameter), each filled with 2.5 kg autoclaved (0.11 Mpa, 121°C, 2 h) soil. The soil for the experiment was collected from a citrus orchard of Yangtze University campus and taxonomically classified as Xanthi-Udic Ferralsols (FAO system). The 60 g inoculum of *F. mosseae* containing sands and spores (23 spores/g) was mixed with 2.5 kg soil at the time of transplanting. Non-AMF treatment also received the same quantity sterilized inoculum and 2 mL inoculum filtrate (25 μm filter) to keep similar microbial communities other than the AM fungus. The strain of the AM fungus, *F*. *mosseae* (Nicol. & Gerd.) Schüßler and Walker (BGC XZ02A), isolated from the rhizosphere of *Incarvillea younghusbandii* in Dangxiong (90^o^45′E and 29^o^31′N, altitude 4 300 msl), Tibet. The AM fungus was propagated with both the identified fungal spores and white clover (*Trifolium repens*) for 16 weeks under potted conditions. The spore density of growth substrate was 23 spores per g, on the basis of wet sieving and decanting method (Gerdemann and Nicolson, 1963) and stereoscopic microscope. The experiment was performed in an environmentally controlled plastic greenhouse (photosynthetic photon flux density 982 μmol/m^2^/s, day/night temperature 27/20°C, and relative humidity 80%) from March 15 to August 1, 2013. The position of pots in the glasshouse was re-randomized at weekly interval in order to expose experimental plants to avail equitable distribution of growing conditions.

### EXPERIMENTAL DESIGN

Experimental treatments consisted of 2 × 2 factorial randomized block design with two soil water regimes (WW, 75% maximum water holding capacity of soil; DS, 55% maximum water holding capacity of soil) and two mycorrhizal inoculations (with or without *F. mosseae*). Each treatment replicated four times carrying a total of 16 pots.

Drought stress started 82 days after transplanting and continued upto 140 days after transplanting. The soil water status in the pots was determined daily through weighing and the amount of water loss was accordingly supplemented in order to maintain soil water status at 6:00 PM every day.

### PLANT OBSERVATIONS AND ANALYSIS

Seedlings were harvested after 58 days of water treatments. The growth related parameters such as plant height, stem diameter, and leaf number per plant were recorded. At harvest, the plants were divided into shoots and roots, and their fresh weights were recorded. Subsequently, the leaves were stored at -80°C for the determination of soluble protein, CaM, H_2_O_2_, and $O_{2}^{•-}$ concentrations and CAT, Cu/Zn-SOD, and Mn-SOD activities.

A number of 1-cm root segments from root tip (30 root segments per treatment) were cleared by 10% (w/v) KOH and stained with 0.05% (w/v) trypan blue (Phillips and Hayman, 1970). The AM colonization was observed using LEICA DME bio-microscope and expressed as the percentage of the colonized root lengths against the observed root lengths. RWC of fourth fully expanded leaf from top was measured according to the method of Bajji et al. (2001). H_2_O_2_ concentration was determined according to Velikova et al. (2000). A 0.2 g fresh leaf sample was homogenized with 5 mL 0.1 % (w/v) trichloroacetic acid in an ice bath and centrifuged at 12,000 × *g* for 15 min. Then 1 mL supernatant was mixed with 1 mL 10 mM potassium phosphate buffer (pH 7.0) and 2 mL 1 M KI, following which absorbance was recorded at 390 nm.

Fresh leaf samples (0.2 g) were homogenized in 5 mL of 0.1 M phosphate buffer (pH 7.8) and centrifuged at 4,000 × *g* for 10 min at 4°C. The supernatant was used to determine soluble protein, $O_{2}^{•-}$ and CAT. Leaf soluble protein concentration was determined using bovine serum albumin as the standard (Bradford, 1976). Leaf $O_{2}^{•-}$ concentration was measured using the method as described by Wang and Luo (1990). The 0.5 mL of the supernatant was mixed with 0.5 mL of 50 mM phosphate buffer (pH 7.8) and 0.1 mL of 10 mM hydroxylamine chloride reaction. After 1 h reaction at 25°C, the mixture was added to another mixture containing 1 mL 17 mM sulfanilamide and 1 mL 7 mM α-naphthylamine at 25°C for 20 min, followed by determination of absorbance at 530 nm.

Catalase activity was performed as per the procedure described by Goldblith and Proctor (1950). The reaction mixture included 2.5 mL enzyme extract and 2.5 mL of 0.1 M H_2_O_2_. After incubation at 30°C for 10 min, 2.5 mL of 10% H_2_SO_4_ was added to stop the recation, and 0.1 M KMnO_4_ was used to titrate the residual H_2_O_2_ until a purple color persisted for at least 30 s. CAT activity was expressed as mg H_2_O_2_/g FW/min. While, Cu/Zn-SOD and Mn-SOD activities were measured using the ELISA (A001-2, Nanjing Jiancheng Bioengineering Institute, Nanjing, China) according to ELISA guide. The CaM concentration was assayed using the Plant CaM ELISA Kit (YAD-001, Beijing Dingguochangsheng Biotechnology Co., Ltd., Beijing, China) in terms of the user guide of ELISA.

### STATISTICAL ANALYSIS

Data (means ± SE, *n* = 4) were statistically analyzed by the two-factor ANOVA with SAS 8.1 software (SAS Institute Inc., Cary, NC, USA), and the Duncan’s multiple range tests were used to determine the significance of the treatments at the *P* < 0.05 level.

## RESULTS AND DISCUSSION

### ROOT MYCORRHIZAL COLONIZATION

**FIGURE 1:**
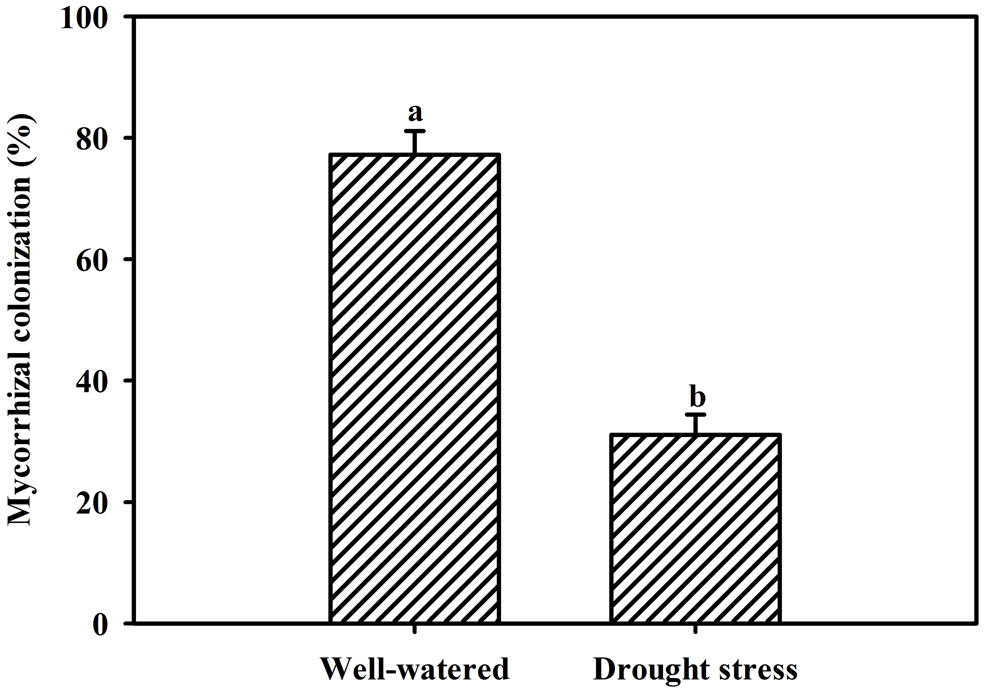
**Root AM colonization of trifoliate orange seedlings by *Funneliformis mosseae* under well-watered (WW) and drought stress (DS) conditions.** Data (means ± SD, *n* = 4) followed by different letters above the bars among treatments indicate significant differences at the 5% level.

Vigor of plant growth depends upon the magnitude of root colonization as a result of AMF inoculation. Inoculation with *F. mosseae* induced varying magnitude of root colonization in trifoliate orange seedlings under both WW and DS conditions. However, root colonization under DS conditions, was observed only 31% compared to 77% under WW conditions (**Figure 1**). Mycorrhizal colonization was observed significantly dependent upon interaction effect of water status and AMF (**Table 1**). Moreover, the 58-day DS treatment significantly reduced the root colonization of trifoliate oranges by AMF. The decrease of root colonization under DS is reported in a wide range of crops (Wu et al., 2013), since spore germination and hyphal spread are strongly dependent on soil water status (Huang et al., 2011).

**Table 1:** Significance of the main treatment effects and their interactions based on two-factor ANOVA on tested variables of trifoliate orange (*Poncirus trifoliata*) seedlings grown on well-watered (WW) and drought stress (DS) conditions.

Variable	Main effects		Interaction effects (Water status × AM)
	Water status	AM	
**Biometric parameters**
Mycorrhizal colonization	**	**	**
Plant height	**	**	NS
Stem diameter	**	**	NS
Leaf number per plant	**	**	NS
Shoot fresh weight	**	**	NS
Root fresh weight	**	**	NS
Total fresh weight	**	**	NS
**Physico-biochemical parameters**
Soluble protein	**	**	NS
Leaf RWC	**	**	NS
Cu/Zn-SOD	**	**	NS
Mn-SOD	**	**	**
CAT	**	**	**
CaM	**	**	NS
H_2_O_2_	**	**	NS
$O_{2}^{•-}$	**	**	NS

NS, not significant; *P < 0.05; **P < 0.01.

### PLANT GROWTH

**FIGURE 2:**
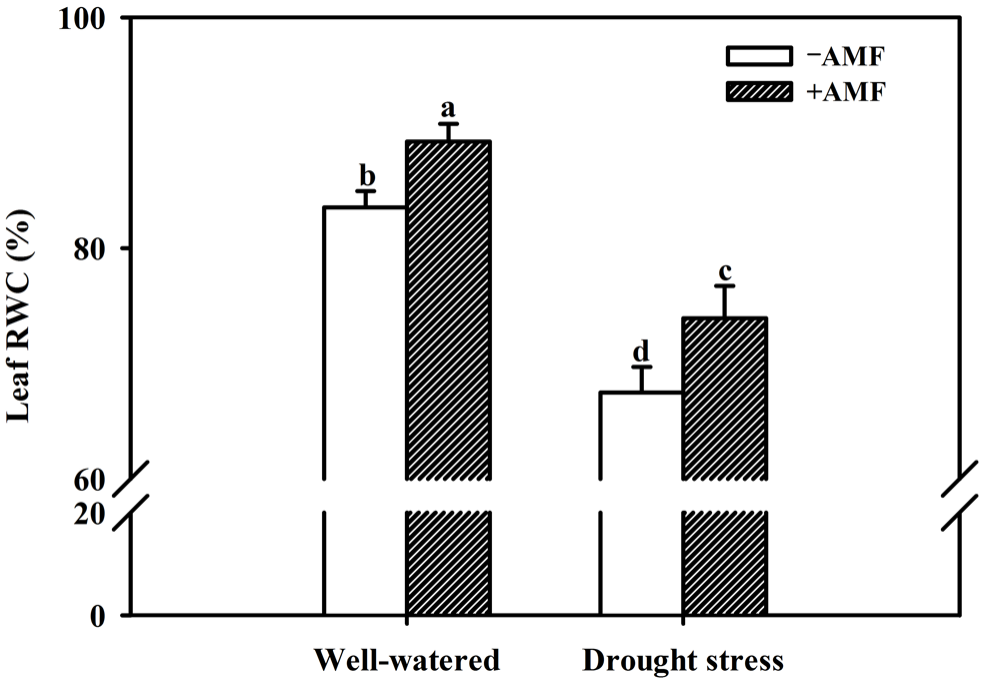
**Effect of an AM fungus (*F. mosseae*) on leaf relative water content (RWC) of trifoliate orange seedlings under under WW and DS conditions.** Data (means ± SD, *n* = 4) followed by different letters above the bars among treatments indicate significant differences at the 5% level.

Mycorrhization significantly improved all the growth related parameters of the trifoliate orange seedlings including plant fresh weight, regardless of soil water status (**Table 2**). Compared with non-AMF control, AMF treatment significantly increased plant height, stem diameter, and leaf number per plant by 21, 5, and 16%, respectively, under WW and by 21, 10, and 9% under DS. Other growth parameters such as shoot, root and total plant (shoot + root) fresh weight in AM seedlings were significantly higher by 27, 23, and 26% over non-AM seedlings under WW. But under DS, the magnitude of response in shoot, root, and total plant fresh weight of AM seedlings compared to non-AM seedlings, was relatively higher by 28, 27, and 28%, respectively. Such a strongly response trend supports that AMF inoculation possessed greater ability to improve plant biomass under DS conditions than under WW conditions. Hence, AMF inoculation significantly increased shoot morphological properties (plant height, stem diameter, and leaf number) and biomass production than non-AMF control, irrespective of whether or not plants are maintained under WW and DS conditions. This is in agreement with the findings of Tian et al. (2013), who reported that AMF colonization significantly enhanced growth of Sacha inchi (*Plukenetia volubilis* L.) seedlings under both WW and DS conditions. The growth improvements induced by mycorrhization under either WW or DS condition have primarily been attributed to an enhancement in absorption capacity of water and nutrients by extraradical hyphae (García et al., 2008; Bárzana et al., 2012). Our observations also showed that AMF colonization significantly increased leaf RWC under both WW as well as DS conditions. Compared with non-AMF-inoculation, AMF inoculation significantly increased leaf RWC by 7 and 10% under WW and DS, respectively (**Figure 2**). Higher RWC in AM seedlings suggested that AM seedlings were capable of absorbing additional water from the rhizosphere or alternatively have greater ability to control water loss through stomatal regulations (Wu and Xia, 2006; Augé et al., 2014).

**Table 2:** Effect of an AM fungus (*Funneliformis mosseae*) on growth of trifoliate orange seedling under WW and DS conditions

Treatments	Growth parameters			Plant fresh weight (g/plant)		
	Plant height (cm)	Stem diameter (mm)	Leaf number per plant	Shoot	Root	Total
WW-AMF	37.3 ± 2.7bc	3.45 ± 0.11b	37 ± 2b	2.37 ± 0.12c	0.92 ± 0.06c	3.29 ± 0.06c
WW+AMF	45.2 ± 1.3a	3.61 ± 0.08a	43 ± 2a	3.01 ± 0.12a	1.13 ± 0.05a	4.14 ± 0.16a
DS-AMF	33.7 ± 2.3c	3.12 ± 0.12c	34 ± 1c	2.12 ± 0.13c	0.79 ± 0.02d	2.91 ± 0.12d
DS+AMF	40.7 ± 3.0b	3.44 ± 0.09b	37 ± 1b	2.72 ± 0.08b	1.00 ± 0.05b	3.72 ± 0.13b

*Data (means ± SD, *n* = 4) followed by different letters among treatments indicate significant differences at 5% level.*

### CHANGES IN CaM AND ANTIOXIDANT ENZYME PROFILE

Mycorrhization associated changes in antioxidant enzymes are widely reported (Hu et al., 2007; Ni et al., 2013). Earlier studies (Ni et al., 2013; Wu et al., 2013) using different citrus species demonstrated that the AMs conferred greater tolerance to plants against soil water deficit through an enhancement in their antioxidant enzyme defense system consequent upon a decrease in level of H_2_O_2_ and $O_{2}^{•-}$. In our studies, the DS induced accumulation of leaf $O_{2}^{•-}$ and H_2_O_2_ concentration, regardless of AMF- or non-AMF-seedlings (**Table 3**). However, compared with non-AMF treatment, AMF inoculation significantly decreased leaf $O_{2}^{•-}$ concentration by 13 and 15% under both the WW and DS conditions, respectively. While AMF-seedlings recorded 19 and 21% lower leaf H_2_O_2_ concentration under WW and DS conditions, respectively, compared to non-AMF-seedlings.

**Table 3:** Effect of an AM fungus (*Funneliformis mosseae*) on Cu/Zn-SOD, Mn-SOD, and CAT activities and soluble protein, $O_{2}^{•-}$ and H_**2**_O_**2**_ concentrations in leaves of trifoliate orange under WW and DS conditions.

Treatments	Soluble protein (mg/g FW)	Antioxidant enzymes			ROS		
		Cu/Zn-SOD (μg/mg protein)	Mn-SOD (μg/mg protein)	CAT (mg H_2_O_2_/min/g FW)		$O_{2}^{•-}$(μmol/g FW)	H_2_O_2_ (μg/g FW)
WW - AMF	22.23 ± 1.67bc	1369 ± 162a	1290 ± 68a	2.21 ± 0.34c		0.23 ± 0.02b	116.0 ± 3.1c
WW + AMF	26.71 ± 1.77a	1481 ± 79a	1426 ± 70a	7.31 ± 0.34a		0.20 ± 0.02c	93.9 ± 13.1d
DS - AMF	21.25 ± 1.50c	942 ± 146b	300 ± 123c	1.62 ± 0.33d		0.30 ± 0.01a	180.4 ± 15.8a
DS + AMF	24.17 ± 0.24b	1275 ± 167a	823 ± 172b	4.25 ± 0.44b		0.25 ± 0.00b	142.1 ± 12.1b

*Data (means ± SD, *n* = 4) followed by different letters among treatments indicate significant differences at 5% level. ROS stands for reactive oxygen species.*

Arbuscular mycorrhizal fungi inoculation was associated with increased soluble protein concentration and CAT activity in leaves, irrespective of soil water status (**Table 3**). AMF-seedlings recorded 20 and 14% higher soluble protein concentration under WW and DS, respectively, in leaves of the plant. While, leaf CAT activity as result of AMF inoculation increased by 231 and 162% under WW and DS, respectively. According to the results of Tian et al. (2013), inoculation with AMF increased CAT activity of *Plukenetia volubilis* plants under DS, thus reducing both accumulation of H_2_O_2_ and oxidative damage to lipids. Another study by Ni et al. (2013) showed significantly higher leaf SOD and root CAT activity in mycorrhizal citrus tangerine seedlings as compared with non-mycorrhizal seedlings under DS conditions. Our study further showed that amongst SODs, leaf Cu/Zn-SOD, and Mn-SOD activities under mycorrhization remained unchanged under WW conditions, but significantly increased by 35 and 174% under DS conditions, respectively, as compared with non-mycorrhization (**Table 3**). These results suggested that AMF inoculation conferred significantly greater magnitude of increase in Cu/Zn-SOD and Mn-SOD activities under DS than WW conditions. The CAT and Mn-SOD activities were significantly affected by the interactive effect between AM and DS (**Table 1**), implying that the DS treatment profoundly stimulated AMs to trigger the over-expression of SOD isozymes, resulting in a lower accumulation of ROS in leaves. Ruiz-Lozano et al. (2001) found that expression of *Mn-SOD II* gene was increased in mycorrhizal plants under DS. AMF inoculation, hence, increased CAT activity under both WW as well as DS conditions, which expanded the defense capacity to host plant against any possible oxidative damage (Ruiz-Lozano, 2003; Wu et al., 2006b; Huang et al., 2011). Maintaining higher antioxidative enzyme activities provides increased resistance to plant against oxidative damage under DS conditions (Sharma and Dubey, 2005).

**FIGURE 3:**
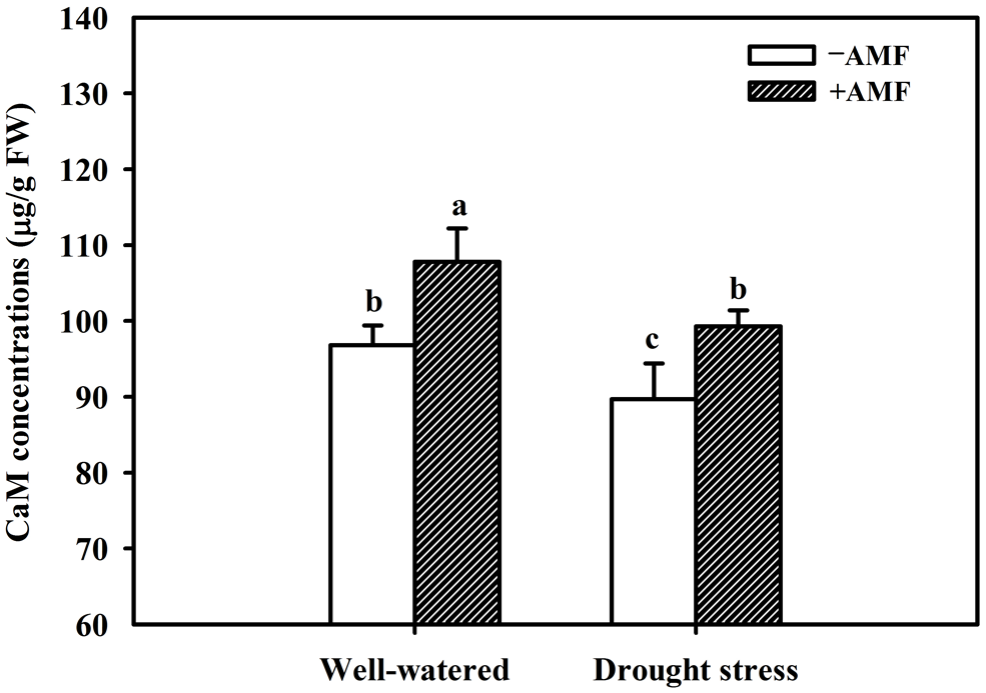
**Effect of an AM fungus (*F. mosseae*) on leaf CaM concentrations of trifoliate orange leaf under WW and DS conditions.** Data (means ± SD, *n* = 4) followed by different letters above the bars among treatments indicate significant differences at the 5% level.

**FIGURE 4:**
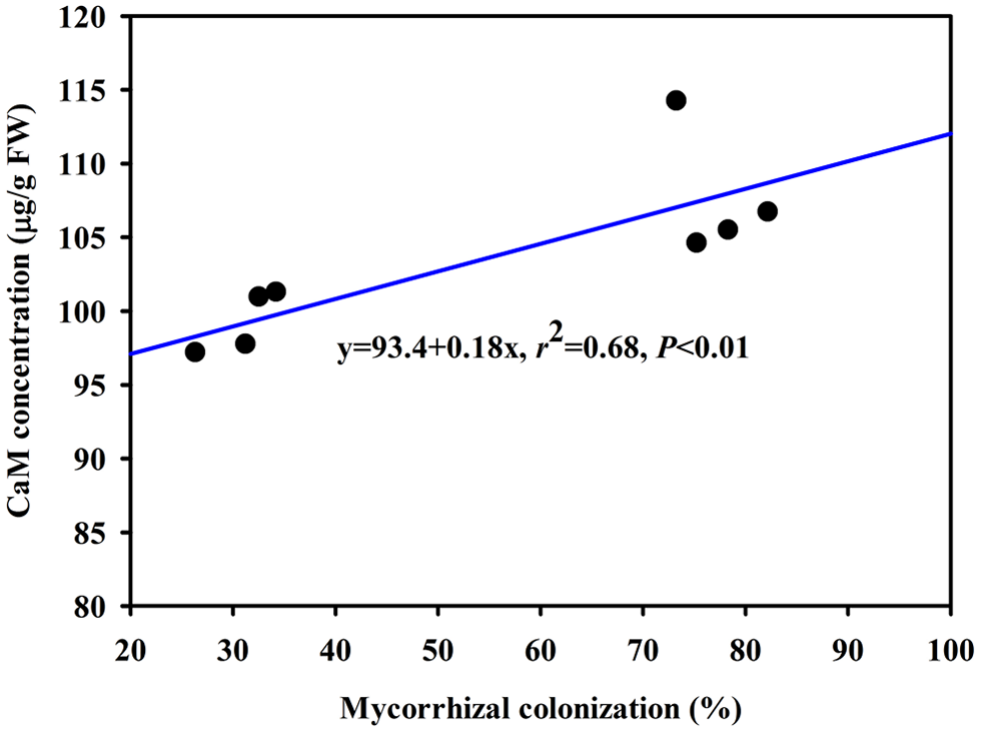
**Line regression between root mycorrhizal colonization and leaf CaM concentration of trifoliate orange inoculated with an AM fungus (*F. mosseae*) under WW and DS conditions (*n* = 8)**.

**FIGURE 5:**
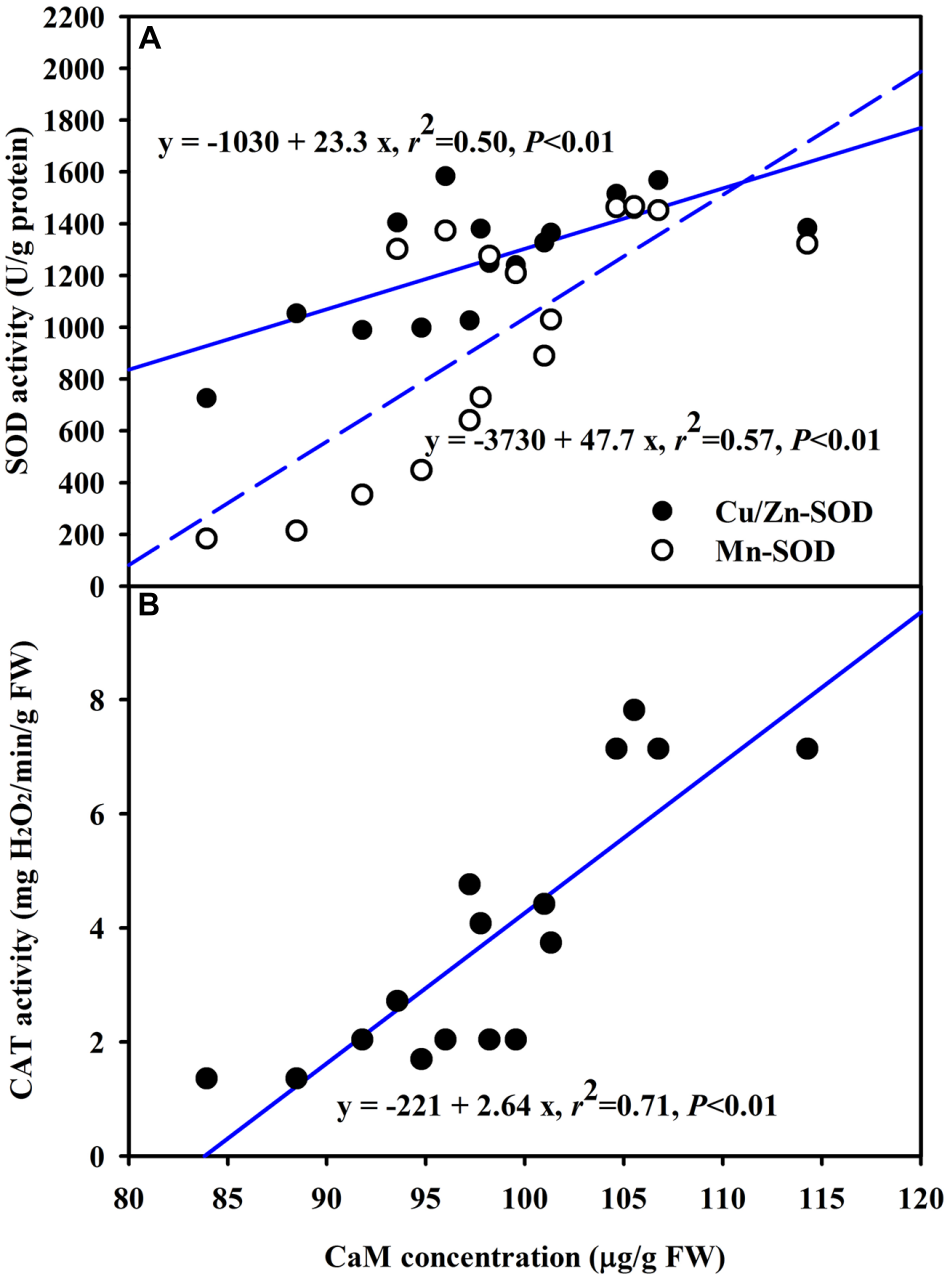
**Line regression between CaM concentration and SODs (Cu/Zn-SOD and Mn-SOD) **(A)** or CAT **(B)** activity in leaves of trifoliate orange inoculated with an AM fungus (*F. mosseae*) under WW and DS condition (*n* = 16)**.

**FIGURE 6:**
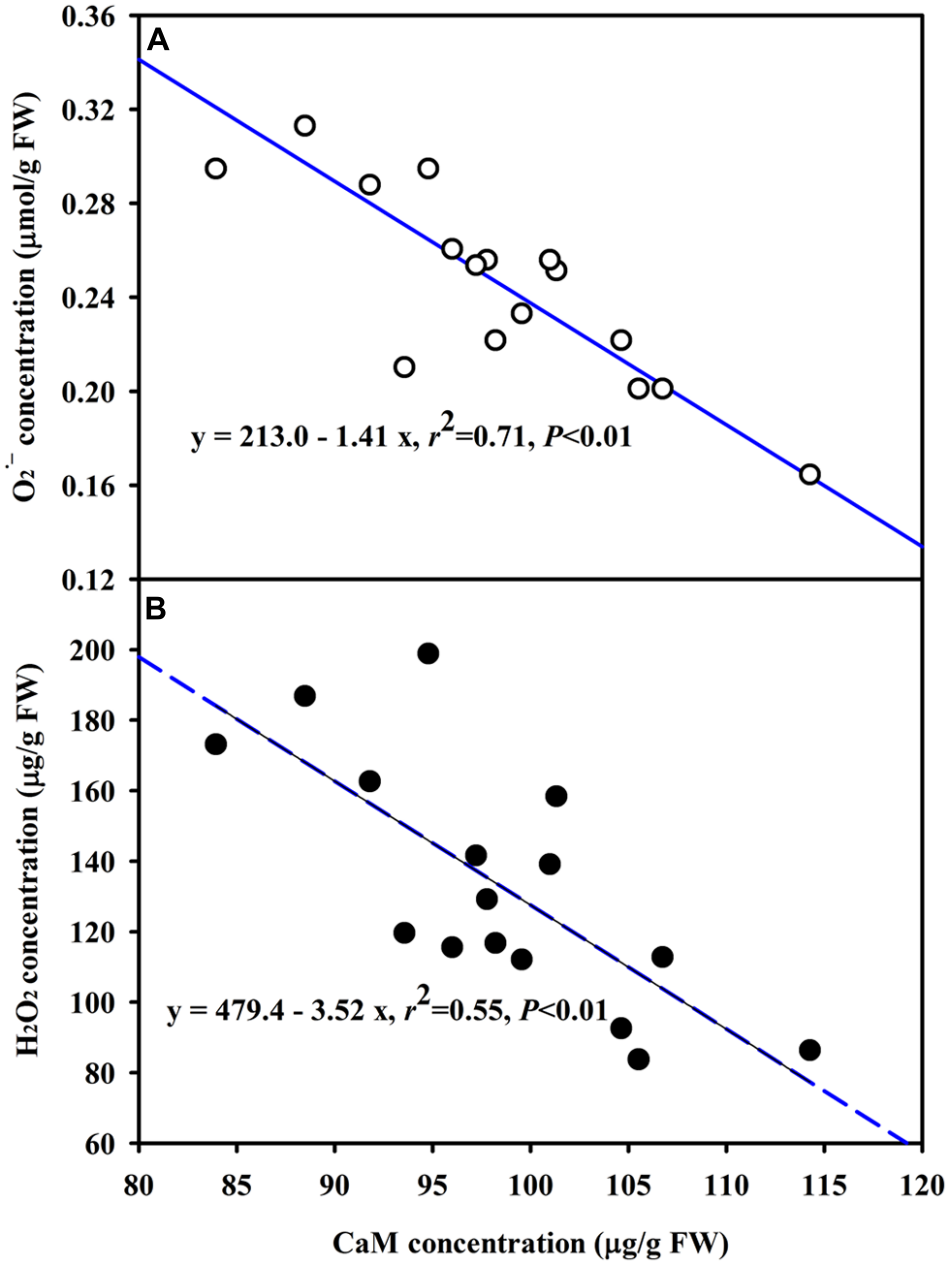
**Line regression between CaM concentration and $O_{2}^{•-}$**(A)** or H_**2**_O_**2**_**(B)** concentration in leaves of trifoliate orange inoculated with an AM fungus (*F. mosseae*) under WW and DS conditions (*n* = 16)**.

The DS treatment induced significant decrease of leaf CaM concentration than WW treatment, irrespective of AMF- or non-AMF-seedlings status (**Figure 3**). However, mycorrhizal inoculation significantly increased leaf CaM concentration by 11% under both WW as well as DS conditions (**Figure 3**). Line regression analysis further supported that leaf CaM concentration was significantly (*P* < 0.01) positively correlated with mycorrhizal colonization (**Figure 4**), suggesting that root AM colonization modulated leaf CaM levels, or CaM as the second messenger involved in root mycorrhizal colonization. There were no significant differences of leaf CaM concentration between AMF-seedlings under DS conditions and non-AMF-seedlings under WW conditions. Leaf CaM concentration was significantly positively correlated with leaf SODs (Cu/Zn-SOD and Mn-SOD; **Figure 5A**) and CAT activity (**Figure 5B**), but negatively correlated with leaf $O_{2}^{•-}$ (**Figure 6A**) and H_2_O_2_ concentration (**Figure 6B**). Our studies, hence, revealed that AMF inoculation induced CaM mediated elevation in antioxidant enzyme activities and reduction in ROS levels. Interestingly, CaM is reported to induce ROS generation as a second messenger mediating signal transduction under various stress conditions (Bowler and Fluhr, 2000; Chen et al., 2004). It seems that CaM is postulated to be of multiple function protein involved in a series of responses, collectively attributing towards plant defense signal network.

An increase of CaM concentration by mycorrhization under both WW and DS conditions would bind more Ca^2+^, thus enhancing the signal strength and accelerating the signal transfer rate to trigger various cellular responses (Shao et al., 2008; Yang et al., 2010). In fact, AMF-seedlings recorded significantly higher root Ca^2+^ influxes under both WW and DS conditions (Zou et al., 2014). Lorella et al. (2007) observed that an AM fungus *Rhizophagus intraradices* early increased the intracellular CaM in soybean cells. These results implied that AM colonization would induce an enhancement of CaM levels, ultimately bringing substantial improvement in capturing the signal strength of plant. Earlier studies (Huang et al., 1995) reported that CaM participated in regulation of SOD activity with SOD as a CaM-binding protein (Gong and Li, 1995). A significantly positive correlation between CaM concentration and CAT activity substantiated that CaM was involved in regulating the CAT activity. Gong et al. (1997) earlier observed that CaM-mediated heat tolerance was associated with an increase in antioxidant system consisting of SOD and CAT activities.

## CONCLUSION

Arbuscular mycorrhizal fungi inoculation, in the present study, significantly improved the growth of trifoliate orange and induced higher CaM synthesis under WW as well as DS conditions. Correlations revealed that AMF-induced CaM concentration mediated SODs and CAT activities aided in scavenging the accumulated ROS, collectively enhancing the drought tolerance of the host plant. Further studies to characterize the nature of CaM (functional significance of CaM) and address the molecular mechanisms of interaction between H_2_O_2_ production and CaM activation, besides how CaM upregulates the antioxidant defense system in the whole process of signal transduction, will provide a better understanding of physiology and biochemistry of changes associated with AMF inoculation.

## AUTHOR CONTRIBUTIONS

Yong-Ming Huang, Qiu-Dan Ni, and Yu Han were involved in acquisition and analysis of data for the work; Qiang-Sheng Wu and Ying-Ning Zou were involved in the design of the work; Yong-Ming Huang and Qiang-Sheng Wu prepared the draft for work; A. K. Srivastava critically revised the whole draft work for important intellectual content. All authors approved the final version.

## Conflict of Interest Statement

The authors declare that the research was conducted in the absence of any commercial or financial relationships that could be construed as a potential conflict of interest.
